# Cognitive Structure of Writing Disorders in Russian: What Would Luria Say?

**DOI:** 10.3233/BEN-2012-119010

**Published:** 2012-05-03

**Authors:** Elena Kozintseva, Anatoly Skvortsov, Anastasia Ulicheva, Anna Vlasova (Zaykova)

**Affiliations:** ^1^The National Research University Higher School of EconomicsMoscowRussia; ^2^The Centre of Speech Pathology and NeurorehabilitationMoscowRussia; ^3^The Russian National Research Medical UniversityMoscowRussia; ^4^Moscow Research Institute of PsychiatryMoscowRussia; ^5^Laboratory for Communication ScienceUniversity of Hong KongHong Kong

**Keywords:** Syndrome approach, cognitive approach, dysgraphia, Russian, Luria, writing

## Abstract

Acquired disorders of writing in the Russian language have been reported for more than a century. The study of these disorders reflects the history of Russian neuropsychology and is dominated by the syndrome approach most notably by the writings of Luria. Indeed, our understanding of acquired dysgraphia in Russian speakers is conceptualized according to the classical approach in Modern Russia. In this review, we describe the classical approach and compare it to the cognitive neuropsychological models of writing disorders that are developed to explain dysgraphia in English and in other Western European languages. We argue that the basic theoretical assumptions of the two approaches – cognitive and classical or syndrome approach – share similarities. It is therefore proposed that identification of acquired cases of dysgraphia in Russian could potentially benefit from taking the cognitive neuropsychological perspective. We also conclude that adopting elements of the syndrome approach would substantially enrich the understanding of acquired dysgraphia since these offer an insight into processes not described in the cognitive neuropsychological approach.

## 1. Introduction

The theory of dynamic and systematic localization of higher mental functions proposed by A.R. Luria dominates Russian neuropsychology in the present day. This reflects the syndrome approach in aphasia originating from the classical writings of Broca, Wernicke and Dejerine. The cognitive neuropsychological approach is the dominant paradigm to explain writing difficulties in English. Although this paradigm is proven to be a useful approach in the assessment and rehabilitation of aphasia, it is not yet being implemented in Russia in the clinic or the laboratory.

Our focus in this paper will be on disorders of writing. One reason for this focus is that reports of acquired dysgraphia in Russian are more common than cases of acquired dyslexia, possibly because of the relatively transparent mappings between print and sound in Russian, with more unpredictable mappings from sound to print needed for writing [25].

In the following sections we will review the theoretical assumptions, structure and clinical implications of the classical approach and the cognitive neuropsychological approach in relation to writing. Then Luria's cognitive structure of writing will be introduced, along with an example of its application to a specific writing task – spontaneous writing and written naming. We will also discuss writing disorders in Russian described by Luria and his followers identifying the aspects of Luria's model that may potentially enrich the cognitive neuropsychological approach.

## 2. The classical approach

The revolution in thinking about aphasia and more specifically acquired dysgraphia took place in the 1970s

ISSN 0953-4180/12/$27.50 © 2012 – IOS Press and the authors. All rights reserved as a consequence of the rapid changes in the fields of experimental and cognitive psychology. Prior to the work of Marshall and Newcombe [40,41], our understanding of writing disorders was characterized by a syndrome approach bound tightly to brain localization. For example, the early writings of Dejerine [17] on alexia without agraphia were influential in behavioral neurology well into the 1960s when Geschwind developed a theory of disconnection in the brain [21]. However, even Dejerine proposed a verbal model to account for these phenomena, highlighting a role for the angular gyrus that became known as the word form area [13]. A multitude of taxonomies for acquired writing disorders were proposed in the 20$^{th}$ century including aphasic agraphia, alexia with agraphia, pure agraphia, apraxic agraphia and collosal agraphia. Each of these syndromes was to become less important to the study of patients with writing disorders, since none of them offered any potential to treat or rehabilitate the increasingly vital skills (spelling, typing) required for employment in the modern age. Moreover, modern neuroimaging studies revealed that the assumed relationships between brain regions such as the visual word form area and spelling ability are either not correct or incomplete [7,43,50,51].

It is instructive to compare syndromes of the classical approach with disorders identified in Russian speakers in the past 100 years. Pure agraphia is a disorder of writing that occurs due to abnormal functioning of the brain and is not accompanied by other disorders of mental function as in many neurological disorders [12,30, 31,52]. Apraxic agraphia is not a specific writing dysfunction and is always accompanied by apraxia. Disorders of writing affect separate letters as graphic symbols that cause illegible writing and the disautomatization of the writing process [38,54]. Aphasic agraphia describes the tendency for a spelling disorder to reflect the features of the oral language disorder. So for example in fluent aphasia a patient may display a tendency to produce written output that is jargon containing letter omissions, substitutions and perseverations, whereas in non-fluent aphasia the patient may not be able to produce a single stroke [38].

## 3. Cognitive neuropsychological approach

The cognitive neuropsychological approach emerged in the latter part of the 20$^{th}$ century and is currently the dominant paradigm used to explain cases of acquired dysgraphia in English and other languages (as can be seen in this Special Issue). In terms of writing disorders, the dual route framework can explain the pattern of errors observed across most cases as a result of damage to one or more pathways mapping phonology to orthography used for normal spelling in children and adults [5,15,16,18,20,22–24,27,45,48,49,53, 55,58–61].

According to Ellis [20], there are two routes available for normal spelling: a lexical route which processes a whole word as a unique lexical entry and a non-lexical route which processes words according to their sub-symbolic (sound) properties. The lexical route requires retrieval of stored phonological and orthographic knowledge of the whole word. A word that is spoken for dictation (orally or in writing) is processed by the phonological input lexicon, which stores representations of word sounds, e.g., syllables. Word form activation is followed by access to the conceptual representations of a word stored in the semantic system. This in turn activates the store of written word forms in the orthographic output lexicon which are used to produce a whole word form via the motor response system that assembles letter representations, case representations and finally a fluent written production. The motor response system can also be accessed via the non-lexical spelling pathway, however this occurs via a conversion of phonemes into graphemes without any contact to the whole word form or semantic system. This conversion takes place via phoneme to grapheme correspondences operating via the nonlexical spelling system. In most Indo-European languages this system can be used to spell nonwords (sound sequences without meaning) as well as regularly spelled words that follow predictable phoneme to grapheme correspondences, e.g., *jar*, although the utility of a non-lexical spelling system has been questioned for other languages, such as Chinese [63]. The process of spelling to sound conversion involves (a) auditory to phonological conversion whereby sounds are broken into their corresponding phonemes and (b) temporary storage in a phonemic buffer for the serial conversion of phonemes into corresponding letter strings. The output from both the lexical and the non-lexical spelling routes feeds into a graphemic buffer which is a short term memory store with direct access to a motor output system for the spelling of words orally, by typing or gesture and in written form. Each of these systems is independent of the other thus allowing a patient to be able to write the name of a picture from visual input but not from dictation and vice versa. In writing, patients can show a discrete set of impairments involving formation of letters with spatially similar letters (b, d, p, q) more easily confused or selective difficulties generating the upper or lower case of a letter (B or b). Cases of mirror writing reflect problems at this peripheral stage of motor output according to the theory.

Acquired *surface dysgraphia* results from damage to the lexical spelling pathway and consequent reliance on phoneme to grapheme conversion for spelling all words. Errors are 'phonological' as they refer to a loss of knowledge about the unpredictable mappings between sound and print in a language with a tendency to apply phoneme to grapheme correspondence rules when spelling e.g. *yacht* spelled as YOT. The most salient feature of surface dysgraphia is better spelling of regular words than irregular words and a tendency to produce regularised versions of irregular spellings [6, 62]. In surface dysgraphia intact nonword spelling is coincident with regularised spelling for irregular words. This is taken as evidence for an impaired lexical route whereas the nonlexical route is preserved. Acquired *phonological dysgraphia* results from damage to the nonlexical spelling pathway and reliance on the whole word forms for spelling. Errors are 'lexical' because they are whole words when nonlexical knowledge is requested, e.g., *nar* spelled as CAR. Phonological dysgraphia is reported to correspond with an impaired ability to spell nonwords to dictation while familiar word spelling is fairly preserved [5,8,52]. Within the dual-route model of spelling phonological dysgraphia is assumed to reflect an impairment to assembly of print from sound via the nonlexical route whilst the retrieval of previously learnt, familiar words via the lexical route is intact.

In the case of acquired *deep dysgraphia*, there is near complete abolition of the nonlexical spelling system and lexical word form system leading to the production of semantic errors, e.g., *symphony* written as ORCHESTRA due to complete reliance on the semantic system for writing all words. The body of evidence supporting this classification emerged from orthographies with mixed (i.e. opaque and transparent) mappings between sound and print such as English and French [6,8,41]. The hallmark of deep dysgraphia is a lexicality effect, i.e. better spelling of words than nonwords, and an imageability effect, i.e. better spelling of high imageable words than low imageable words [8, 26,44]. These impairments are taken as evidence of an impaired nonlexical route together with a preserved semantic system.

*Graphemic buffer dysgraphia* describes a pattern of errors in which graphemic transpositions, e.g., *letar* for later or substitutions, e.g., *leter* for later predominate. These errors are assumed to arise as a result of failure of the buffer to hold sound based information for a sufficient time to allow the assembly of grapheme for motor production. Note that damage to the buffer could lead to errors in oral, typing or written spelling performance [57]. Moreover, buffer damage will affect the spelling of both words and nonwords since the buffer holds information in store from both spelling pathways.

One interesting feature of a buffer dysgraphia is the tendency to preserve the linguistic properties of a word so that a vowel is substituted for another vowel and not a consonant, e.g., *trep* for trip, a letter cluster is substituted for another letter cluster, e.g., *grench* for stench and even a geminate cluster is substituted for another geminate, e.g., *bugger* for butter. Such errors are interesting for a variety of reasons including insights into the mental representation of a whole word in the lexical language system [20]. The multiple levels of cognitive processing that are necessary for spelling and writing likely require more than one brain region for accurate written word production [47].

## 4. Comparing the syndrome and cognitive neuropsychological approaches

The syndrome approach can be contrasted to the cognitive method that appeared around the latter part of the 20$^{\text{th}}$ century. Some writers point out that the different approaches have very similar principles [9], noting that the original Wernicke-Lichtheim model was the first cognitive neuropsychological conceptualization of aphasia [14]. For instance, sensory and motor gestalt centers described by Lichtheim have something in common with lexicons in dual route models. Other authors concentrate on differences between syndrome and cognitive neuropsychological approaches [10,11,46]. In our opinion, the basic theoretical propositions of these two approaches are essentially the same. First, different individuals are assumed to have equivalent mental processes. Second, mental processes such as language and speech are made up of components that are responsible for elementary cognitive operations. Third, different brain lesions can result in partial or total disorder to one of several components of a mental process. Therefore, the structures of a mental process in normal cognitive function and in pathology differ only by the presence or absence of the disordered components resulting in cognitive dysfunction. Finally, dysfunction of one component can lead to a particular difficulty in accomplishing some tasks - for instance speech tasks such as naming, repeating, spontaneous speech and writing disorders.

The key differences between the classical and the cognitive neuropsychological approaches are that (a) syndromes are not stable symptom associations; (b) descriptions of aphasia syndromes are not based on modern linguistic knowledge about language structure; and (c) unambiguous data about the localization of mental processes are largely absent although modern brain imaging methods supposedly offer a clearer window into isomorphism [19]. It is worth noting that in the cognitive approach these problems can be dealt with not a revolutionary reconsideration of the basic theoretical propositions but with a more detailed specification of cognitive processes. For instance these criticisms may be addressed by further study of the essential mental processes. So syndrome instability may be resolved if the symptoms correspond to particular components uncovered through experimental psychology, which classic syndromes did not take into account. Followers of the cognitive approach consider that speech brain localization has to be based on a more detailed understanding of speech structure (problems a and b) than is currently available, as well as on precise investigation of brain localization using neuroimaging methods. In Russia today, behavioral neurologists consider that the cognitive neuropsychological approach to study of language disorders does not require rejection of the main propositions of a syndrome approach but should enhance them. Therefore, data from speech pathology of classic syndromes can be reconsidered in light of the tools offered by modern neuropsychology. Although the views of Russian aphasiologists are not as widely known as Lichtheim or Geschwind, they can be used to enrich our understanding of aphasia in Russian speakers and potentially in other languages.

## 5. Luria's approach

In Russian aphasiology, A.R.Luria was the first scientist to conduct detailed neuropsychological analyses of acquired writing disorders. Luria's ideas about writing structure and dysgraphias are based on examinations of about 800 patients with cognitive disorders resulted from brain injury during World War II [37]. His detailed and extensive observations are described in "The Essays of Writing Psychophysiology" in 1950 [35,36].

Luria reported on writing processes more than reading processes – a fact that reflects the greater prevalence of acquired dysgraphia than dyslexia in Russian, which is likely to be due to the orthographic transparency of the Russian writing system for reading but not for writing [25]. Luria planned to write an analogous paper devoted to the psychological structure of reading but, unfortunately, those plans were not fulfilled. Nevertheless, there is a description of the component structure of writing in Luria's monograph "The Traumatic Aphasia" [37]. Furthermore reports of acquired writing disorders can be found in Luria's classic textbooks [32–34,37] and in papers written by the followers of Luria scientific school in Russia [1,2].

Luria's theory of the dynamic localization of higher mental functions supposes the functions are compound psychological systems consisting of many subcomponents. The same component can be included in the structure of several different mental functions (in Luria's terms a component is named a "link" as if in a chain). Every component is closely connected to the functioning of one specific brain area. If a lesion in such a brain area leads to a dysfunction of a component that is common for several mental functions, this will result in a single neuropsychological syndrome, which includes disorders to a variety of different mental functions. All these disorders can be characterized by one common quality. In this way Luria explains why a local brain lesion may result in a pattern of different disorders in apparently unrelated mental functions for example, speech and praxis, memory, perception simultaneously. At first glance it might be unclear why Luria often associated one component of mental process with several dissimilar functions. In this article we do not refer to this complex issue (see classical Luria's monographs [32–34,37]).

At the same time different subtypes of one disordered mental function can be caused by a dysfunction of different components. On the last point, Luria's tradition is similar to the modern cognitive neuropsychological approach. According to Luria, the structural organization of writing includes some components corresponding to a single function, and other components that are shared with other verbal and even nonverbal processes. All components are linked together in a way that allows these processes to function correctly.

Luria's ideas about the structure of writing are to a greater extent psychological rather than linguistic. Although Luria did not address the question of compatibility of the model with other languages himself, it might be suggested that the Luria's syndrome approach can be applied not only to Russian but also to other languages with a different degree of consistency of sound-to-letter correspondences, e.g., English [56].

## 6. Cognitive components of writing in Luria's framework

According to Luria, one of the cognitive components necessary for writing is the *regulative component*. This process has four functions: 1) to formulate and maintain the motive and goal (the desirable result of an activity) to direct the behaviour of an individual in a meaningful way; 2) to regulate the writing process according to this meaning; 3) to control the results of the writing process according to the intended meaning; and 4) the conceptual-semantic system serves the understanding of the general meaning of a text and formulating and maintaining this meaning for writing. The goal of writing can be formulated by the patient or given externally. The activity of the prefrontal region of the human brain is assumed to be the biological substrate of successful engagement of this cognitive component.

The next cognitive component of writing is *programming of the utterance*. This is responsible for creating a plan, which includes formulating the main segments of the concept and sequencing these segments. This planning can be performed with help of inner speech. The neuroanatomical basis of it is assumed to be the left posterior frontal area.

Another component of writing is *successive organization of language units*. This has three functions: a) syntactic organization of the utterance (Broca's area is a neurological substrate for this operation); b) kinetic articulatory programming and c) kinetic programming of graphemes. The latter is required for the sequential organization of phonemes and graphemes.

*Visual-gnostic perception* is also an important component of writing. Every grapheme can be represented as a set of visual features that must be organized to form a complex structure. Thus, the visual-gnostic component operates with graphemes as complex visual gestalts. The visual-gnostic ability is assumed to be a function of secondary regions in the left occipital cortex. According to the cognitive neuropsychological approach, such a component allows phoneme-to-grapheme conversion as is assumed to be necessary for spelling via the non-lexical pathway.

Another multifunctional component of writing is called *simultaneous synthesis*. This mechanism also has several functions. First, the lexical-semantic system is involved in simultaneous synthesis. This system performs the operations within the system of concepts (mental lexicon), which are organized as a simultaneous net. It is needed for word actualization and perception. This system is also a fundamental component of many dual route models of spelling, which assume that word forms and also word meanings are represented as a neural network of interconnected nodes in a parallel distributed framework [33,37]. Second, simultaneous synthesis involves spatial gnosis. Spatial gnosis is used to orient the graphemes within spatial coordinates. The cognitive neuropsychological approach refers to this as the peripheral writing system, which is specific to the execution of writing and is not relevant to the processes used for oral spelling or typing. Third, quasi-spatial synthesis is used for syntactic decoding of logical-grammatical quasi-spatial language constructions. It is assumed necessary for understanding the meaning of a text, which is desirable for dictation. Luria suggested that this component of simultaneous synthesis is based on the activity of left parietal-temporal-occipital area. Although the dual route model of spelling assumes a conceptual system, it has not developed beyond the single word level and thus Luria's approach offers potential for further development in this component of language processing and central disorders of spelling.

We have to mention another important stage of writing according to Luria – *sound-letter analysis and synthesis*. This stage is complex and consists of several interrelated cognitive components performing simultaneously. In essence, sound-letter analysis and synthesis consists of two units labelled phonological and articulatory by Luria. Each unit can be divided into two operations. The phonological unit is divided into a) phonological analysis and b) phonological synthesis. These operations are highly interconnected and perform simultaneously, because phonological analysis cannot be accomplished without phonological synthesis. On one hand, the writer cannot compose a phonological image of a word without images of separate phonemes. Phonological analysis is responsible for differentiating phonemes. On the other hand, the essential features of a particular phoneme depend on phoneme position in the word, i.e. on surrounding phonemes. So phonological synthesis is required for combining isolated speech sounds into complex units, such as syllables and words. The phonological unit including analysis and synthesis is assumed to be a function of left temporal lobe.

The articulatory unit is the second necessary part of sound-letter analysis and synthesis. It is divided into a) kinesthetic articulatory analysis and b) kinetic articulatory synthesis. The relations between these operations are the same as for phonological analysis and synthesis. During kinesthetic articulatory analysis the writer articulates aloud or silently the word to write and defines the sound compounds of the word. In this case the speech sounds are coded for their articulatory kinesthetic schemes. Luria supposed, such kinesthetic articulatory analysis takes place in every writing act and during the acts of speech perception, but the pronunciation of words usually has reduced inner form. The brain substrate of this component is the left inferior parietal region.

As for the phonological unit, kinesthetic schemes of speech sounds cannot be detected separately. This is because the features of each articulation depend on surrounding phonemes.[1] To synthesize the kinesthetic schemes of phonemes kinetic programming or kinetic articulatory synthesis is needed. This process organizes kinesthetic schemes of speech sounds into a successive sequence. Broca's area is assumed by Luria to be the necessary neural substrate of this component.

It is important to note that Luria assumes the articulatory and phonological sound-letter analysis and synthesis systems to be two complementary mechanisms. They work hand in hand and are both necessary for accomplishing sound-letter analysis and synthesis. Acoustic-verbal memory is also a necessary component of writing. This component maintains speech sounds during the writing and reading process. This operation is assumed to require the left medial temporal gyrus.

As can be seen, Luria's framework includes some components that are ignored in the cognitive neuropsychological approach, for instance, the regulative component responsible for the ongoing control and regulation of the writing process and successive organization of language units and sound-letter analysis and synthesis. Moreover, components in Luria's model may be associated with the elements of cognitive models. For example, Luria's phonological analysis would allow phoneme-to-grapheme conversion in a nonlexical pathway of the neuropsychological approach. The functions of both conceptual-semantic and lexical semantic systems seem to be performed by the semantic system in the cognitive neuropsychological approach. The phonological buffer also relies on acoustic-verbal memory [57].

In the next section we discuss the structure of a specific writing task, spontaneous writing and naming as an example of implementation of Luria's notions about writing.

[1] In Russian the features of a phoneme are subject to change depending on the surrounding phonemes and its position in a word. For example, a phoneme [b] typically represented by the letter "б" becomes palatalized ([b']) before front vowels, such as [i], [e]; and becomes unvoiced at the end of a word ([p]). It could be hypothesized that the involvement of phonological and kinetic articulatory synthesis in more orthographically consistent languages (such as Spanish or Italian) may be reduced.

## 7. Cognitive structure of spontaneous writing and naming

The first stage of spontaneous writing is formulating the motive and goal of writing and concept. Afterwards, programming of the utterance takes place. Subsequently the lexical-semantic system actualizes the meanings of words and lexical entries that have to be organized syntactically. Then a complex operation of sound-letter analysis and synthesis should be executed. The processes of speech memory play a role in maintaining the speech sounds or sound images of words for future needs. At the next stage the visual-gnostic component activates gestalts of graphemes in order to perform a phoneme-to-grapheme transformation. Spatial organization of graphemes follows. The final step of the writing process is sequencing the graphemes during the motor act of writing. It should be mentioned that if the goal is to write not a sentence but a single word the procedure bypasses the stages of programming and syntactic organization of the utterance. Writing can also be accomplished through a different mechanism called an ideographic pattern. This mechanism performs an automatic direct connection between the lexical-semantic system and kinetic sequencing of graphemes. Through this pathway highly automatized words can be written.

One distinctive feature of Luria's view on spontaneous writing is the presence of continuous online regulation and control of writing performed by the regulative component. This takes place at every stage of the writing process. The regulation and control functions serve to map writing processes to the goal and the concept formulated earlier using numerous feedback connections. The structure of spontaneous writing is illustrated in Fig. 1.

## 8. Disorders of writing

Writing disorders can emerge when one or more cognitive components of writing are impaired. As a result, any writing task that recruits an impaired component will disintegrate in a specific manner according to the Luria framework. These patterns are summarized below.

A disruption of the regulative component will manifest as a syndrome of disordered planning, regulation

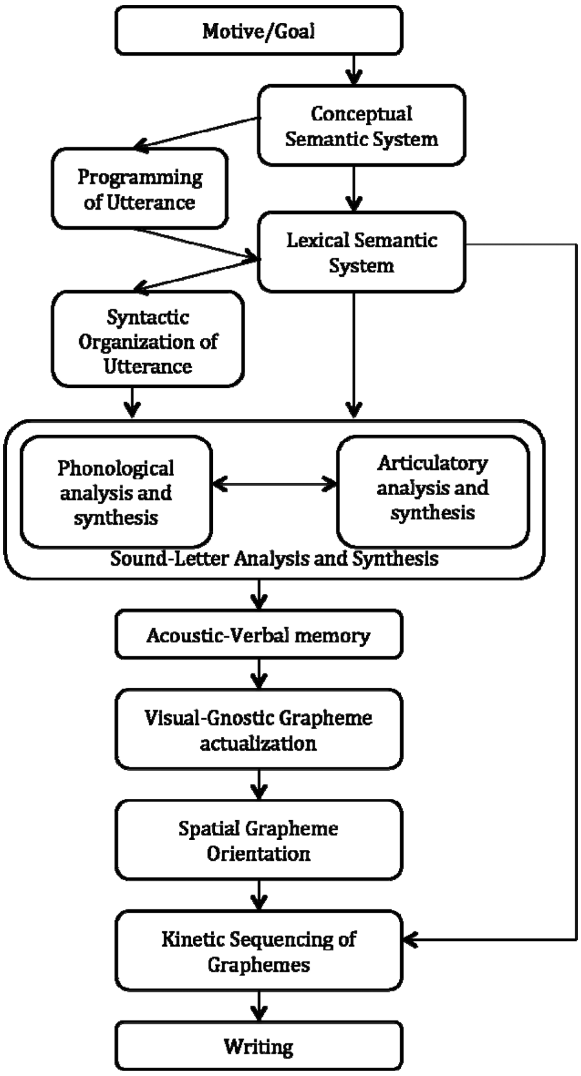

Fig. 1. Cognitive structure of spontaneous writing and written naming in Luria's approach.
The following connections were omitted for better visual representation: each one of the elements {writing, sound-letter analysis and synthesis, syntactic organization of utterance, lexical semantic system} is connected to {motive/goal and conceptual semantic system}; each one of the components {kinetic sequencing of graphemes, spatial grapheme orientation, visual-gnostic grapheme actualization, acoustic verbal memory} is connected to {conceptual semantic system and motive/goal}.

and control of behavior. The goal and the concept that direct writing can be simplified or inadequate. This reflects impairment to the online regulation and control of these processes, which would be subordinated to the goal and concept-semantic system in normal written output.

If programming of an utterance is impaired, difficulties in external expression of the speech intention can be observed. The patient usually knows what he wants to write in general, but has difficulties in formulating the text in full detail. This type of agraphia is called *dynamic*.

A disorder of the lexical-semantic system is the cause of *amnestic* agraphia, when a patient has difficulties with word actualization, manifesting in form of semantic paraphasias (substitutions).

Impairment to the phonological analysis and synthesis has similar implications, but on a smaller grain size level. Literal paraphasias in letters, which share phonological features (for instance, "b" and "p"), is the main symptom of this type of agraphia called *sensory* agraphia. This writing disorder is one of the characteristics of sensory aphasia. Impairment of kinesthetic articulatory analysis and synthesis is the cause of *kinesthetic motor* aphasia. Substitutions of letters similar in articulation may be observed in written word output, for example, "m" and "b".

Another form of motor writing disorder identified by Luria is *efferent motor* agraphia. It is a characteristic of efferent motor aphasia. Agrammatism and disturbance of the kinetic successive sequencing of articulatory schemes of speech sounds and graphemes is the core impairment in this disorder. It usually manifests in repetitions, omissions and displacement of letters.

*Acoustic-mnestic* agraphia, that accompanies acoustic-mnestic aphasia, emerges if speech memory is disturbed. It results mainly in difficulties in maintaining phonological information. Impairments with phoneme-to-grapheme transformation are considered to be an autonomous writing disorder. A patient has knowledge of phonemes and graphemes independently of each other, but has difficulties in establishing correspondences between them.

An impairment of a component responsible for visual-gnostic analysis manifests itself as a problem in actualizing and composing the gestalts for graphemes. A patient can substitute letters, which are close in visual-gnostic features, for example, "*к*" [k] and "х" [kh]. Luria referred to this type of writing disorder as an *optical* agraphia and considered it to be a part of a more general visual-gnostic syndrome.

Other patients can change letters similar in spatial features, for instance, left-right or top-down letter orientation problems (when a patient writes "b" instead of "d"). This is regarded as a central symptom of spatial agraphia and a symptom of a general syndrome of spatial disorders.

The cognitive neuropsychological approach takes into account the evidence that there are distinct pathways for oral and written spelling [57]. In Luria's tradition spelling (sound-letter analysis) and writing are also distinguished. If sound-letter analysis becomes impaired, the patient would spell and write words making the same errors. If the sound-letter analysis system is intact, some letter substitutions may nevertheless occur in writing. This phenomenon is explained through disruption of phoneme-to-grapheme conversion (note that grapheme-to-phoneme conversion may be intact so the patient may become aware of a mistake). Similarly, there are cases when sound-letter analysis is impaired but writing is intact. This is called *ideographic writing* (Fig. 1; the connection between the lexical semantic system and kinetic sequencing of graphemes).

The following tasks are used to assess dysgraphia in Russia: 1) automatized writing (name, home town); 2) writing single letters from dictation; 3) pointing to letters; 4) sound-letter analysis: a) oral spelling; b) identifying the number of letters in a word; c) assembling words from single letters written on separate cards; 5) writing words, phrases, sentences to dictation; 6) writing with the tongue clamped (to reduce inner pronunciation); 7) spontaneous writing. It should be noted however that the assessment in Luria's approach is based on a syndrome approach. This means that each symptom is interpreted in the context of other symptoms. Thus, for an effective assessment other mental functions should be taken into account, for instance, in order to diagnose dysgraphia testing oral speech is also essential.

It is likely that there are unreported cases of acquired dysgraphia in Russian speaking adults and children that reflect the patterns of deep, phonological and surface dysgraphia in other languages. Russian is a transparent script from orthography to phonology (feedforward) because there are no inconsistencies in the mappings between print and sound (with only a few exceptions: for instance, a consonant "ч" that is usually read as [ch] is pronounced as [sh] in two words "что" (*what*) and "конечно" (*of course*)).[2] However, as in other Indo-European languages with a writing system that is mostly feedforward consistent (Italian, Slovak, Spanish), such languages are not necessarily transparent for writing from dictation [16]. The Russian writing system is highly feedback inconsistent, that is many sounds and sound combinations can be written in a number of different ways (for example, [ш] occurring at the end of the word can be written as "ш", "щ", "шь" or "щь").

[2]Unlike many transparent languages, however, the pronunciation of a letter in Russian depends on its position and context and is governed by a set of rules [25].

## 9. Conclusion

The influence of orthographic transparency on acquired dysgraphia in different languages has become an important theoretical issue in the field. Languages with transparent writing systems are of interest because it was assumed that disorders of reading and writing would not exist [4]. However, there are reports of developmental dysgraphia [3] and acquired dysgraphia in Italian [39,42] as well as reports of acquired dysgraphia in Spanish [28] and Turkish [48]. Therefore, there are strong reasons to predict these patterns in acquired and developmental cases in Russian.

Reports of acquired dysgraphia in Russian are plentiful in the literature [32–37]. However, they all reflect one theoretical perspective only – the syndrome approach. The present review has shown that this tradition has many basic similarities with cognitive approach. So these two traditions could enrich each other and this integration could be useful for the treatment and rehabilitation of acquired dysgraphia in adults and children with developmental dysgraphia. However, adapting the cognitive neuropsychological approach to Russian speakers raises issues that need attention before a paradigm shift can occur in Russia toward a focus in assessment on the impact of orthographic transparency on writing disorders based on established methods [29].

Luria's views on writing disorders differ from the cognitive neuropsychological paradigm in a remarkable way. The first distinction is about the different component composition of cognitive processes. The second difference is that Luria assumes a component is associated to a distinct brain area. Moreover, most of the components for writing are not specific to one mental process, but are necessary for other verbal and nonverbal functions as are the processes of attention and memory that are becoming increasingly important in cognitive neuropsychological models. This results from Luria's conceptions about syndrome structure and the principles of his theory of dynamic and systematic localization of higher mental functions.

Probably the most important difference between the two approaches is that in cognitive models the terms "structural component" and "the function of the component" are not distinguished. One structural unit corresponds to one function; in other words, it is associated with just one function. Therefore, damage of this structural component leads to a loss of that function. In Luria's theory one structural component can have several functions included in different stages of processing. If this structural component is disturbed, this results in simultaneous loss of all its functions, and this complex of dysfunctions forms a neuropsychological syndrome. It should be noted however that a structural component does not assume a specific brain anatomical structure but rather a component of a mental process. For instance, if a structural component such as the "successive organization of language units" is defective, this will lead to a simultaneous loss of all functions such as successive articulatory sequencing, successive sequencing of graphemes and syntactic organization of the utterance. It is interesting that these three functional components, which are provided by one structural component, are involved at the different stages of writing. It can be seen that in Luria's theory one structural component does not correspond to a particular stage of a mental process as in cognitive models. One structural component can operate at several stages and, on the contrary, one stage of a mental process can include simultaneous involvement of several different structural components (as can be seen in sound-letter analysis and synthesis).

Despite the fact that Luria's views belong to the syndrome approach, writing structure in his theories comply with the logic of the cognitive neuropsychological approach. This is a result of Luria's notion that a mental function consists of structural components playing their own specific roles and damage of each component disturbs mental process in a specific way.

## Acknowledgements

The article was supported by the Basic Research Program at the National Research University Higher School of Economics (HSE, Russia) in 2012.
